# Clinical outcomes in people with diabetes‐related foot infections: Analysis from a limb preservation service infection database

**DOI:** 10.1002/jfa2.12040

**Published:** 2024-07-09

**Authors:** Matthew Malone, Emma Bergamin, Kenshin Hayashi, Saskia Schwarzer, Hugh G. Dickson, Namson Lau, Lawrence A. Lavery, Robert J. Commons

**Affiliations:** ^1^ South West Sydney Limb Preservation and Wound Research Sydney New South Wales Australia; ^2^ Infectious Diseases and Microbiology School of Medicine Western Sydney University Sydney New South Wales Australia; ^3^ High Risk Foot Service Liverpool Hospital Sydney New South Wales Australia; ^4^ Ingham Institute of Applied Medical Research Liverpool New South Wales Australia; ^5^ Ambulatory Care and PIXI Liverpool Hospital Sydney New South Wales Australia; ^6^ Department of Diabetes and Endocrinology Liverpool Hospital Sydney New South Wales Australia; ^7^ Department of Plastic Surgery University of Texas Southwestern Medical Center Dallas Texas USA; ^8^ Global Health Division Menzies School of Health Research Charles Darwin University Darwin Northern Territory Australia; ^9^ General and Subspecialty Medicine Grampians Health Ballarat Ballarat Victoria Australia

**Keywords:** diabetic foot infection, osteomyelitis, outcomes, skin and soft tissue infection

## Abstract

**Background:**

Diabetes‐related foot infections are common and represent a significant clinical challenge. There are scant data about outcomes from large cohorts. The purpose of this study was to report clinical outcomes from a large cohort of people with diabetes‐related foot infections.

**Methods:**

A tertiary referral hospital limb preservation service database was established in 2018, and all new episodes of foot infections were captured prospectively using an electronic database (REDCap). People with foot infections between January 2018 and May 2023, for whom complete data were available on infection episodes, were included. Infection outcomes were compared between skin and soft tissue infections (SST‐DFI) and osteomyelitis (OM) using chi‐square tests.

**Results:**

Data extraction identified 647 complete DFI episodes in 397 patients. The data set was divided into two cohorts identifying each infection episode and its severity as either SST‐DFI (*N* = 326, 50%) or OM (*N* = 321, 50%). Most infection presentations were classified as being moderate (PEDIS 3 = 327, 51%), with 36% mild (PEDIS 2 = 239) and 13% severe (PEDIS 4 = 81). Infection resolution occurred in 69% (*n* = 449) of episodes with failure in 31% (*n* = 198). Infection failures were more common with OM than SST‐DFI (OM = 140, 71% vs. SST‐DFI = 58, 29%, *p* < 0.00001). In patients with SST‐DFI a greater number of infection failures were observed in the presence of peripheral arterial disease (PAD) compared to the patients without PAD (failure occurred in 30% (31/103) of episodes with PAD and 12% (27/223) of episodes without PAD; *p* < 0.001). In contrast, the number of observed infection failures in OM episodes were similar in patients with and without PAD (failure occurred in 45% (57/128) of episodes with PAD and 55% (83/193) of episodes without PAD; *p* = 0.78).

**Conclusions:**

This study provides important epidemiological data on the risk of poor outcomes for DFI and factors associated with poor outcomes in an Australian setting. It highlights the association of PAD and treatment failure, reinforcing the need for early intervention to improve PAD in patients with DFI. Future randomised trials should assess the benefits of revascularisation and surgery in people with DFI and particularly those with OM where outcomes are worse.

**Abbreviations**
ABPI: ankle brachial pressure index
DFI: diabetic foot infection
ESR: erythrocyte sedimentation rate
HbA1c: glycated haemoglobin
HRFS: high risk foot service
IWGDF: international working for the diabetic foot
OM: osteomyelitis
PAD: peripheral arterial disease
PEDIS: Perfusion, Extent, Depth, Infection and Sensation (PEDIS) Classification System
SST‐DFI: skin and soft tissue infection in the diabetic foot
WIfI: wound, Ischaemia, and foot infection classification system

## 1 INTRODUCTION

Foot infections in persons with diabetes are predominantly associated with ulcerations of the skin and represent a major causal pathway to lower extremity amputation. Factors which increase the risk of foot ulceration have been well reported in the literature and include complications of diabetes such as peripheral neuropathy with associated loss of protective sensation, peripheral arterial disease (PAD), altered foot architecture or deformity, immunological disturbances and often trauma [1]. Once the protective barrier of the skin is breached, microorganisms colonise the exposed skin and soft tissue structures before inducing infection, commonly termed “diabetes‐related foot infection or DFI” [2]. A percentage of infections progress to osteomyelitis (OM), involving osseous/bone structures of the foot. Thus, DFI presentations can be categorised by the site of anatomical involvement as the skin and soft tissue, bone, or both.

Diabetic foot infections are present in more than 40% of patients with foot ulcers [3, 4]. The management of DFIs requires a systematic approach, including diagnosing the pathology, obtaining appropriate specimens for culture and sensitivities to identify pathogens, selecting appropriate antibiotic therapy and rapid decision‐making regarding surgical intervention or hospitalisation. This decision‐making process should include the patient and their carers as part of shared‐care decision‐making. The International Working Group on the Diabetic Foot (IWGDF) has published evidence‐based guidelines on the diagnosis and treatment of foot infection in persons with diabetes mellitus to aid clinicians [2].

Despite global guidelines on management, there is a paucity of data on clinical outcomes in people with DFI. This creates a challenge in benchmarking management and clinical outcomes and when comparing between geographical locations. This lack of data contributes to challenges in designing robust clinical trials as well as hinders the identification of pertinent clinical questions or gaps in the evidence that may be amenable to research.

The purpose of this study is to report the clinical outcomes from a cohort of persons with DFI presenting to a large tertiary multidisciplinary high‐risk foot service (HRFS). Results aim to inform our understanding of DFI outcomes to better understand factors that can be modified to improve outcomes and to assist with designing future clinical trials.

## 2 RESEARCH DESIGN AND METHODS

Liverpool Hospital is an acute quaternary referral hospital in Liverpool, Sydney. The local government area of South‐Western Sydney where Liverpool Hospital is situated is culturally and linguistically diverse and encompasses suburbs with some of the highest rates of diabetes in Australia [5]. The limb preservation service is an outpatient clinic at Liverpool Hospital with 9 staff podiatrists plus consulting physicians and surgeons. Data for this study was collected prospectively from the creation of a limb preservation service DFI database in 2018, using a customised electronic database (REDCap) [6] with approval of the SWSLHD Human Research Ethics Committee (2020/ETH02129). The REDCap software includes the web server and database server, the communication between those two servers, and the communication of the web server with the REDCap end‐user. The security and storage of data from REDCap is managed centrally by the user's individual host institution IT infrastructure (in this case South West Sydney Local Health District).

Patients over the age of 18 presenting with a new DFI episode were included in the database if they provided informed consent and permission for their de‐identified data to be included. Consecutive patients from August 2018 to May 2023 with complete data on infection episodes were included in the analysis. The following data were collected: demographics, medical history, laboratory results, ulcer history (anatomical location, grade, wound metrics and duration of ulcer [new ulcer defined as <4 weeks and chronic ulcer >4 weeks]), infection history (PEDIS grade, classification and duration at presentation), microbiology (culture method, culture results and susceptibilities), antibiotic management (empiric vs. targeted, type of antibiotic, route of administration and duration of antibiotic therapy) and clinical outcomes (infection resolution or infection failure, surgical debridement with or without joint resection, minor amputation, major amputation and mortality). The data were analysed by creating two cohorts based on the anatomical level of infection: skin and soft tissue DFI and OM, either with or without skin and soft tissue DFI. Each cohort was analysed independently and in combination. The primary variables of interest were infection outcome, defined as infection resolution or treatment failure. Secondary variables of interest included comorbidities, ulcer history, infection history, microbiology and antibiotic management.

Treating clinicians undertook the clinical grading of skin and soft tissue DFI and OM (and diabetic foot ulcers) using two validated classification systems, which incorporates wound observations, ischaemia and foot infection measures (abbreviated as WIfI) [7]. This classification system utilises aspects of the IWGDF guidance document for the diagnosis, classification and management of DFI [2] and serves as a template to aid in the relevant collection of clinical, radiological and laboratory data.

Patients with confirmed clinical infection were followed regularly at weekly or second weekly review appointments. During these appointments, the infection and ulcer status were re‐evaluated using the above‐mentioned classification systems for specific endpoints; infection resolution was defined as the clinical cure of infection in response to antibiotic treatment, denoted by no further observable clinical signs or symptoms of infection, ulcer healing or non‐healing. Treatment failure was defined as ≥2 signs or symptoms of infection substantially worsening from baseline and a requirement to alter therapy, which could involve either escalation to a broader spectrum antibiotic, or switching from oral to parenteral therapy, or any unplanned additional antibiotic therapy, surgery or hospitalisation required to control the index infection.

Statistical analysis was performed using SPSS version 29. Chi‐square tests were conducted to test for differences between cohorts with skin and soft tissue infections (SST‐DFI) alone and osteomyelitis with or without associated skin and soft tissue infection (OM). For all comparisons and modelling, the level of significance was set at *p* < 0.05.

## 3 RESULTS

### 3.1 Patient demographics and general infection history

A total of 658 DFI episodes were recorded between August 2018 and May 2023, including 11 active infection episodes with incomplete data which were excluded, leaving 647 DFI episodes in 397 patients. There were 326 (50%) SST‐DFI episodes and 321 (50%) OM episodes (Table 1). The median number of infection episodes per patient was one (range 1–8 DFI episodes per patient; 1 DFI episode = 268 patient, 2 DFI episodes = 85 patients, 3 DFI episodes = 30 patients, 4 DFI episodes = 14 patients, 5 DFI episodes = 5 patients, 6 DFI episodes = 7 patients, 8 DFI episodes = 1 patient).

**TABLE 1:** Demographics and clinical characteristics are reported at the infection episode level.

Patient characteristics	SST‐DFI episodes *N* = 326	Osteomyelitis episodes *N* = 321	Total *N* = 647
Mean age (years)	61.3 (SD = 12.2)	64.5 (SD = 13.2)	62.9 (SD = 12.8)
Gender (Male/Female)	233 (71%)/93 (29%)	243 (78%)/78 (22%)	475 (73%)/172 (27%)
Type 1 or type 2 diabetes	22 (7%)/304 (93%)	19 (6%)/302 (94%)	42 (6%)/605 (94%)
Comorbidities			
Loss of protective sensation	261 (97%)	227 (97%)	622 (96%)
Obesity	125 (38%)	10 (33%)	230 (36%)
Ischaemic heart disease	94 (29%)	91 (28%)	185 (29%)
Hypertension	255 (78%)	253 (79%)	508 (78%)
Chronic kidney disease stage 4	23 (7%)	18 (6%)	41 (6.3%)
Stroke/Transient ischaemic attack	15 (5%)	26 (7%)	41 (6.2%)
Claudication	44 (13%)	42 (13%)	86 (13%)
Rest pain	62 (19%)	75 (23%)	137 (21%)
History of open bypass for lower extremity arterial disease	7 (2%)	19 (6%)	26 (4%)
History of percutaneous transluminal angioplasty for lower extremity arterial disease	61 (19%)	75 (23%)	136 (21%)
WIfI ischaemia grade			
Grade 0 ABPI >0.8, toe pressure >60mmHg	196 (54%)	169 (46%)	365 (56%)
Grade 1 ABPI 0.6–0.79, toe pressure 40–59 mmHg	41 (48%)	45 (52%)	86 (13%)
Grade 2 ABPI 0.4–0.59, toe pressure 30–39 mmHg	20 (38%)	32 (62%)	52 (8%)
Grade 3 ABPI <0.4, toe pressure <30 mmHg	28 (47%)	32 (53%)	60 (9%)
No ABI data	42 (50%)	41 (50%)	83 (13%)
Infection grade at baseline (number of episodes)			
PEDIS 2	170 (100%)	N/A	170 (26%)
PEDIS 3	123 (38%)	275 (62%)	398 (62%)
PEDIS 4	33 (42%)	46 (58%)	79 (12%)

Patient/Clinician history of the infection	Estimated duration of index SST‐DFI at baseline presentation (in days)	Estimated duration to diagnosis of OM from baseline (in weeks)	
PEDIS 2	5 days (SD = 4.6)	2.7 weeks (SD = 4.3)	N/A
PEDIS 3	3.6 days (SD = 4.5)	2 weeks (SD = 3.2)	N/A
PEDIS 4	3.4 days (SD = 5)	0.3 weeks (SD = 1.3)	N/A
Total	4.67 days (SD = 4.75)	2 weeks (SD = 3.4)	N/A

*Note*: There was 647 infection episodes recorded in 397 patients. NB: PEDIS 1 (Uninfected)—No systemic or local symptoms or signs of infection; PEDIS 2 (Mild)—Infected: At least two of these items are present Local swelling or induration, Erythema >0.5 but <2 cm around the wound, Local tenderness or pain, Local increased warmth Purulent discharge; PEDIS 3—Infection with no systemic manifestations and involving: Erythema extending ≥2 cm from the wound margin, and/or tissue deeper than skin and subcutaneous tissues (e.g., tendon, muscle, joint, and bone); PEDIS 4—Any foot infection with associated systemic manifestations (of the systemic inflammatory response syndrome [SIRS]); Add (O)—Infection involving bone (osteomyelitis).

Most infection presentations were classified as moderate (PEDIS 3 = 327, 51%), followed by mild (PEDIS 2 = 239, 36%), with severe infections being the least common presentation (PEDIS 4 = 81, 13%). All infection episodes were associated with a foot ulcer, with 40% (*n* = 258) occurring in a new ulcer and 60% (*n* = 389) in a chronic ulcer. The estimated duration of infection symptoms reported by patients presenting with SST‐DFI at baseline was 4.7 days (SD = 4.8 days). Of the 321 people with OM, 63% (*n* = 202) were suspected or diagnosed at baseline and 37% (*n* = 119) were diagnosed later in their episode of care (mean time before diagnosis = 5.2 weeks, SD 4.2).

### 3.2 Foot ulcer assessment

Wound ischaemia foot infection (WfI) Grade 1 ulcers were the most common presentation in the dataset (*n* = 288, 45%), and this was driven predominantly by their presence in SST‐DFI (*n* = 214, 74%) (Supporting Information S1). Grade 2 ulcers were the second most common (*n* = 273, 42%), followed by Grade 3 ulcers (*n* = 86, 13%). Foot ulcers occurred on the toes in 51% (*n* = 330) of people, forefoot in 34% (*n* = 223), midfoot in 8% (*n* = 53) and hindfoot in 7% (*n* = 41) (Supporting Information S2).

### 3.3 Biochemical markers

Blood tests for biochemical markers were collected in 555 of 647 DFI episodes at baseline (Supporting Information S3). An analysis on all episodes was undertaken in addition to stratification by SST‐DFI or OM. ESR was higher in osteomyelitis compared with SST‐DFI (57 mm/hr [SD 35] vs. 47 mm/hr [SD 30]; *p* < 0.01). Patients with PEDIS grade 4 infections also presented with higher HbA1C levels at baseline (*p* < 0.0001) (Supporting Information S4).

### 3.4 Infection outcomes

Infection resolution was recorded in 448 (69%) DFI episodes and infection failure in 193 (31%) DFI episodes (Table 2 and Supporting Information S5). SST‐DFI episodes were more commonly associated with infection resolution as compared to OM (SST‐DFI resolved = 268, 82% vs. OM resolved = 181, 56%; *p* = 0.0001). Furthermore, we observed increasing PEDIS grades being associated with infection failure in both SST‐DFI (*p* = 0.0001) and OM (*p* = 0.0001; Table 2). The most notable contributor to this statistic were those observed in PEDIS grade 4 infections.

**TABLE 2:** Infection outcomes for 647 DFI episodes by infection type and PEDIS grade.

	Total	Resolved	Failed	*p* value
Total	647	448 (69%)	199 (31%)	
SST DFI	326	268 (82%)	58 (18%)	<0.0001
OM	321	181 (56%)	140 (44%)	
Total infection outcomes by PEDIS grade (*n* = 647)				
PEDIS 2	243	211 (87%)	32 (13%)	<0.0001
PEDIS 3	325	211 (65%)	114 (35%)	
PEDIS 4	79	26 (33%)	53 (67%)	
SST DFI outcome by PEDIS grade (*n* = 326)				
PEDIS 2	170	157 (92%)	13 (8%)	<0.0001
PEDIS 3	123	95 (77%)	28 (23%)	
PEDIS 4	33	16 (48%)	17 (52%)	
DFO outcome by PEDIS grade (*n* = 321)				
PEDIS 3 (O)	275	171 (58%)	104 (42%)	<0.0001
PEDIS 4 (O)	46	10 (22%)	36 (78%)	

*Note*: *p* values were calculated for each grouping using either a 2 × 2 or 3 × 2 contingency table (chi square).

Peripheral arterial disease (PAD) was present in 36% (*n* = 231) of all episodes (SST‐DFI = 103, 45% and OM = 128, 55%). In patients with SST‐DFI, a greater number of infection failures were observed in the presence of PAD compared to patients without PAD (failure occurred in 30% (31/103) of episodes with PAD and 12% (27/223) of episodes without PAD; *p* < 0.001) (Table 3 and Supporting Information S6). In addition, Infection failure increased in relation to a greater ischaemia grade (Ischaemia grade 0 = 12% failure, grade 1 = 14% failure, grade 2 = 39% failure and grade 3 = 50% failure) (Table 4 and Supporting Information S7). PAD was present in 40% (*n* = 128) of episodes with OM; however, the number of observed infection failures were similar in patients with and without PAD (failure occurred in 45% (57/128) of episodes with PAD and 55% (83/193) of episodes without PAD; *p* = 0.78). When PAD was stratified by ischaemia grade the percentages of infection failures increased (in relation to PEDIS 0 and 1), and were higher in Grade 2 and 3 OM episodes (Ischaemia grade 0 = 43% failure, grade 1 = 29% failure, grade 2 = 61% failure and grade 3 = 51% failure) (Table 4 and Supporting Information S7).

**TABLE 3:** Chi square correlation (2 × 2 contingency table) between SST‐DFI and OM resolution or failure and PAD.

	SST DFI resolved (*n* = 268, 82%)	SST DFI failure (*n* = 58, 18%)	*p* value	OM resolved (*n* = 181)	OM failure (*n* = 140)	*p* value
PAD (*n* = 231, 36%)	72 (70%)	31 (30%)	<0.0001	71 (55%)	57 (45%)	0.78
No PAD (*n* = 416, 64%)	196 (88%)	27 (12%)		110 (57%)	83 (43%)	

**TABLE 4:** WIfI grading scale of DFI episodes stratified by infection type.

Ischaemia grade	Total (*n* = 647)	SST DFI resolved	SST DFI failure	*p* value	OM resolved	OM failure	*p* value
0	416 (64%)	196 (88%)	27 (12%)	<0.0001	110 (57%)	83 (43%)	<0.01
1	105 (17%)	43 (86%)	7 (14%)		39 (71%)	16 (29%)	
2	61 (9%)	14 (61%)	9 (39%)		15 (39%)	23 (61%)	
3	65 (10%)	15 (50%)	15 (50%)		17 (49%)	18 (51%)	

Ischaemia grade	ABI	Ankle systolic pressure (mmHg)
0	≥0.80	>100
1	0.6–0.79	70–100
2	0.4–0.59	50–70
3	≤0.39	<50

*Note*: The table reports the 4 × 2 contingency table (chi square value) for both SST‐DFI and OM.

There were 380 interventions undertaken for the 198 DFI episodes associated with infection failure. The most common intervention was percutaneous transluminal angioplasty with or without stenting (*n* = 138, 36%), followed by minor amputation (*n* = 118, 31%) and surgical debridement (*n* = 87, 23%). Major amputations and open bypass surgery were uncommon and accounted for only 11 (3%) and 26 (8%), respectively, of all interventions (Table 5). Interventions were more common in SST‐DFI and osteomyelitis episodes of PEDIS grade 3 and 4 (Table 5).

**TABLE 5:** 198 DFI episodes with infection failure as an outcome required 380 surgical interventions.

	Surgical debridement	Minor amputation	Major amputation	Angioplasty +/− stenting	Open bypass	All procedures
Total	87 (23%)	118 (31%)	11 (3%)	138 (36%)	26 (8%)	380 (100%)
SST‐DFI	30 (34%)	31 (26%)	6 (54%)	61 (44%)	0 (0%)	128 (34%)
OM	57 (66%)	87 (74%)	5 (46%)	77 (66%)	26 (100%)	252 (66%)
Infection failure requiring surgery in SST‐DFI by PEDIS grade						
PEDIS 2	5 (6%)	7 (6%)	0 (0%)	25 (18%)	0 (0%)	37 (10%)
PEDIS 3	14 (16%)	14 (12%)	3 (27%)	25 18 (%)	0 (0%)	56 (15%)
PEDIS 4	11 (13%)	10 (8%)	3 (27%)	11 (8%)	0 (0%)	35 (9%)
Infection failure requiring surgery in DFO by PEDIS grade						
PEDIS 3 (O)	36 (41%)	65 (55%)	2 (19%)	63 (46%)	20 (77%)	186 (49%)
PEDIS 4 (O)	21 (24%)	22 (19%)	3 (27%)	14 (10%)	6 (23%)	66 (17%)

*Note*: Patients may have more than one surgical intervention per episode recorded.

## 4 DISCUSSION

This prospective cohort study of 397 people and 647 episodes from Liverpool Hospital in Sydney is one of the largest cohorts of people with DFI reported in Australia. It found close to a third of infection episodes failed treatment, with this rate rising to almost half of episodes associated with OM. Peripheral arterial disease and a higher PEDIS grade were also associated with increased treatment failure.

Despite management in a tertiary center with a multidisciplinary HRFS, 18% of SST‐DFI episodes and 44% of OM episodes ended with failure of infection treatment in this study. Variable risks of infection recurrence have been reported. A study of patients undergoing surgical debridement or partial amputation for DFI in Switzerland found treatment failure occurred in 20%–31% of episodes [8]. In contrast, a study assessing management of OM with conservative surgery found recurrent infection to occur in just 4.6% of patients. However, with 25% of these patients requiring reoperation due to persistent infection and 43% developing re‐ulceration, part of the variation in outcomes likely relates to a difference in the definitions of failure [9]. In this current study, treatment failure was defined as any clinical infection requiring escalation in antibiotic management or unplanned surgery or hospitalisation.

Consistent with the current study, OM has been associated with reduced resolution of infection and an increased risk of minor and major amputations. This highlights the importance of accurate diagnosis of OM and effective treatment. The current study found ESR to be higher in infections associated with OM, compared with SST‐DFI; however, the increase was relatively small (57 vs. 47 mm/hr). These results are consistent with a 2019 systematic review which found a high ESR (≥70 mm/hr) to have reasonable sensitivity and specificity for predicting OM [10]. A more recent systematic review suggested procalcitonin was more discriminatory to identify OM accurately [11]. Procalcitonin was not tested in the current study and is not readily available at all centers in Australia. The current IWGDF recommendations recommend combining biomarkers or using them with other diagnostic tests such as probe‐to‐bone testing or plain X‐rays to diagnose OM [2].

Historically, OM was treated primarily with surgery. However, recent retrospective evidence and a small randomised controlled study have suggested that antibiotic therapy alone may be as effective as surgery in some patients with forefoot OM [12, 13]. Based on these results, the 2023 IWGDF infection guidelines include a conditional recommendation to consider antibiotic therapy without surgery for diabetic foot OM where there is no need for immediate surgery or drainage to control infection, there is no PAD and there is no exposed bone. Thus, management without surgery still requires rapid evaluation of the infected limb and its arterial supply together with close involvement and decision‐making of surgeons.

PAD is associated with an increased risk of infection in foot ulcers [14], in addition to reduced healing and higher risk of amputation [15]. The negative prognosis of PAD highlights the importance of rapidly assessing and treating it, where possible, in all patients with DFI. The recommendation against using antibiotic therapy alone for OM in patients with PAD highlights the strong association of this condition with poor outcomes in DFI. This was reflected in the current study, where PAD was associated with increased treatment failure in SST‐DFI. Surprisingly we observed comparable treatment failure rates in OM episodes in patients with and without PAD.

To explain this observation, we postulate potential confounding areas. Firstly, we acknowledge the potential limitations around the operational definitions we used in the study. One of the biggest unmet needs in evaluating people with diabetes is good tools to measure PAD. The high prevalence of calcification raises questions about the reliability of the conventional segmental arterial Doppler studies to diagnose PAD [16]. We may simply have used the wrong operational definition(s) or the wrong technology to evaluate perfusion. Our findings may also be attributed to a selection bias effect. Patients with OM and prominent signs of ischaemia would be more likely to have immediate surgical intervention, removing them from the cohort of patients with OM who are available to be managed as outpatients with antibiotic therapy. However, patients with minor degrees of ischaemia of the skin in addition to infection would be managed on an expectant basis, and this might then result in more failures. Further, the operational definition we used to confirm OM in addition to infection resolution and failure were based primarily on clinical, radiographic and laboratory markers and not bone biopsy or histopathology. This may increase the likelihood of an incorrect diagnosis.

Secondly, there may be other variables which have a stronger causal relationship to infection failure or resolution that supersede PAD in patients with OM. Our research group were the first to demonstrate that biofilms (slow growing bacteria) were the main microorganism phenotypes in OM and that their presence may explain why non‐surgical treatment of DFO (with systemic antibiotic therapy) may not resolve some chronic infections [17]. Lastly, the blood supply to the skin and soft tissue versus bone are different. The vascular tree and requirements of oxygen for skin‐soft tissue maintenance versus bone is more expansive, thus skin and soft tissue is likely to be more susceptible to changes in both micro‐ and macrovascular disease when compared to a localised section of bone (which relatively smaller by size and homoeostasis requirements) [18]. This may potentially have a greater impact on the success of a therapy, for example, the reduced bioavailability of systemic antibiotics in soft tissues [19].

The study was limited by its observational nature, with an inability to assess interventions in a randomised or blinded framework. Similarly, the heterogeneous nature of the study population and the pragmatic approach to assessment of outcomes without adjusting for confounders means that outcomes may not be consistent with results of a randomised trial and are more representative of real‐world data. In addition, some people had multiple DFI episodes which may lead to some bias when assessing baseline characteristics of episodes. Given the HRFS at Liverpool Hospital does not routinely manage cellulitis without wounds, the results cannot be generalised to this population.

This study provides further important epidemiological data on the risk of poor outcomes for DFI and factors associated with poor outcomes in an Australian setting. In particular, it highlights the association of PAD and treatment failure, reinforcing the need for early intervention to improve PAD in patients with DFI. Future randomised trials should assess the benefits of revascularisation and surgery in people with DFI and particularly those with OM where outcomes are worse.

## AUTHOR CONTRIBUTIONS

**Matthew Malone**: Conceptualization; investigation; methodology; formal analysis; writing – original draft preparation; writing – review & editing. **Emma Bergamin**: Project administration; investigation; data curation; formal analysis. **Kenshin Hayashi**: Data curation; investigation. **Saskia Schwarzer**: Supervision; data curation; investigation; writing – review & editing. **Hugh G. Dickson**: Conceptualization; methodology; formal analysis; writing – original draft preparation; writing – review & editing. **Namson Lau**: Conceptualization; methodology; writing – review & editing. **Lawrence A. Lavery**: Supervision; methodology; writing – review & editing. **Robert J. Commons**: Formal analysis; methodology; writing – review & editing.

## CONFLICT OF INTEREST STATEMENT

The author declares that there is no conflict of interest that could be perceived as prejudicing the impartiality of the research reported.

## ETHICS STATEMENT

De‐identified data for this study was collected prospectively with approval of the SWSLHD Human Research Ethics Committee (2020/ETH02129). Verbal consent for the collection of de‐identified data was obtained from patients.

## Supporting information

Supporting Information S1

## Data Availability

The data that support the findings of this study are available from the corresponding author upon reasonable request.
