# Supplementary material for: Clinical outcomes in people with diabetes‐related foot infections: Analysis from a limb preservation service infection database
Source: J Foot Ankle Res. 2024 Jul 9;17(3):e12040. doi: 10.1002/jfa2.12040 (PMC11633327; doi:10.1002/jfa2.12040)
Supplement: Supplementary file 1 — Supporting Information S1 [file JFA2-17-e12040-s001.docx]

Supplement

Supplement 1. Wifi ulcer grading with overlapping infection grading

|  | **Grade 1 Ulcers** | **Grade 2 Ulcers** | **Grade 3 Ulcers** |
| --- | --- | --- | --- |
| **Total (n= 647)** | 288 (45%) | 273 (42%) | 86 (13%) |
| **PEDIS 2 total (n=245)** | 170 | 63 | 12 |
| **PEDIS 3 total (n=324)** | 97 | 177 | 50 |
| **PEDIS 4 total (n= 78)** | 21 | 33 | 24 |
| **Total SST DFI (n= 326)** | 214 (66%) | 80 (25%) | 32 (9%) |
| **PEDIS 2 SST DFI (n=170)** | 138 | 26 | 6 |
| **PEDIS 3 SST DFI (n=123)** | 63 | 45 | 15 |
| **PEDIS 4 SST DFI (n=33)** | 13 | 9 | 11 |
| **Total DFO (n=321)** | 74 (23%) | 193 (60%) | 54 (17%) |
| **PEDIS 2 DFO (n=75)** | 32 | 37 | 6 |
| **PEDIS 3 (n=201)** | 34 | 132 | 35 |
| **PEDIS 4 DFO (n=45)** | 8 | 24 | 13 |

Supplement 2. Anatomical site of infected ulcer. Number of infections resolved and ulcers not healing. Anatomical location of non-healing ulcers identifies heels and forefoot ulcers are challenging ulcers to heal aside from the managing infection. In total, 35% (226) of DFI episodes were associated with non-healing ulcers. Percentages of non-healing ulcers were calculated from within groups i.e. toes = 91 un-healed of 330 ulcers with DFI = 28%.

|  | **Toes** | **Forefoot** | **Midfoot** | **Heel** |
| --- | --- | --- | --- | --- |
| **Total (n= 647)** | 330 (51%) | 223 (34%) | 53 (8%) | 41 (7%) |
| **PEDIS 2 total (n=245)** | 149 | 72 | 11 | 13 |
| **PEDIS 3 total (n=324)** | 161 | 109 | 28 | 26 |
| **PEDIS 4 total (n=78)** | 20 | 42 | 14 | 2 |
| **Total SST DFI (n=326)** | 146 (45%) | 128 (39%) | 32 (10%) | 20 (6%) |
| **PEDIS 2 SST DFI (n=170)** | 92 | 60 | 11 | 7 |
| **PEDIS 3 SST DFI (n=123)** | 48 | 46 | 16 | 13 |
| **PEDIS 4 SST DFI (n=33)** | 6 | 22 | 5 | 0 |
| **Total DFO (n=321)** | 184 (57%) | 95 (29%) | 21 (7%) | 21 (7%) |
| **PEDIS 2 DFO (n=75)** | 57 | 12 | 0 | 6 |
| **PEDIS 3 DFO (n=201)** | 113 | 63 | 12 | 13 |
| **PEDIS 4 DFO (n=45)** | 14 | 20 | 9 | 2 |

|  | **Toes** | **Forefoot** | **Midfoot** | **Heel** | **Total** |
| --- | --- | --- | --- | --- | --- |
| **Total number of ulcers at infection baseline** | 330 (51%) | 223 (34%) | 53 (8%) | 41 (7%) | 647 (100%) |
| **Infection resolved and ulcers not healing** | 91 (28%) | 97 (43%) | 19 (36%) | 19 (46%) | 226 (35%) |

Supplement 3. Biochemical markers from peripheral blood tests in 555 DFI episodes. Reported as mean and standard deviation.

|  | **WCC**  **(x10^9^/L)** | **ESR (mm/hr)** | **CRP**  **(mg/L)** | **Neutrophils**  **(x10^9^/L)** | **HbA1c**  **(%)** | **HbA1C**  **(IFCC)** |
| --- | --- | --- | --- | --- | --- | --- |
| **All (n=647)** | 10.4  (SD= 5.6) | 53  (SD= 32.7) | 59  (SD= 78.6) | 7.4  (SD= 3.9) | 8.7  (SD=2.1) | 71.8  (SD= 23) |
| **PEDIS 2 SST DFI (n=245)** | 9.1 (SD=2.7) | 43 (SD=29) | 21 (SD=31) | 6.1 (SD=2.2) | 8.7 (SD=2) | 72 (SD=21) |
| **PEDIS 3 SST DFI (n=324)** | 10.8 (SD=9.1) | 52 (SD=30) | 58 (SD=73) | 7.2 (SD=3.3) | 8.7 (SD=2.5) | (72 (SD=27) |
| **PEDIS 4 SST DFI (n=78)** | 14.9 (SD=6.6) | 57 (SD=28.5) | 186 (SD=103) | 12.3 (SD=51.) | 10.3 (SD=2.6) | 89 (SD=29) |
| **All SST DFI (n=326)** | 10.5 (SD=6.7) | 47 (SD=30) | 56 (SD=80) | 7.3 (SD=3.7) | 8.9 (SD=2.3) | 74 (SD=25) |
| **PEDIS 3 DFO (n=276)** | 10 (SD3.2) | 53 (SD=33.2) | 38 (SD=47) | 6.5 (2.9) | 8.5 (SD=2.0) | 70 (SD=21) |
| **PEDIS 4 DFO (n=45)** | 14.3 (SD=6.2) | 82.8 (SD=20) | 172 (SD=103) | 11.6 (SD=6.1) | 9.3 (SD=1.7) | 79 (SD=19) |
| **All DFO (n=321)** | 10.4 (SD=4.4) | 57 (SD=35) | 62 (SD=77) | 7.5 (SD=4.2) | 8.5 (SD=1.8) | 70 (SD=20) |
| **P value (95% CI)**  ***Comparing all SST-DFI to all DFO in a t-test** | .93 (-.9 to .97) | .01 (-17.8 to -1.6) | .3 (-19 to 7.4) | .5 (-.9 to .45) | .06 (-.01 to .77) | .09 (-.6 to 8.1) |


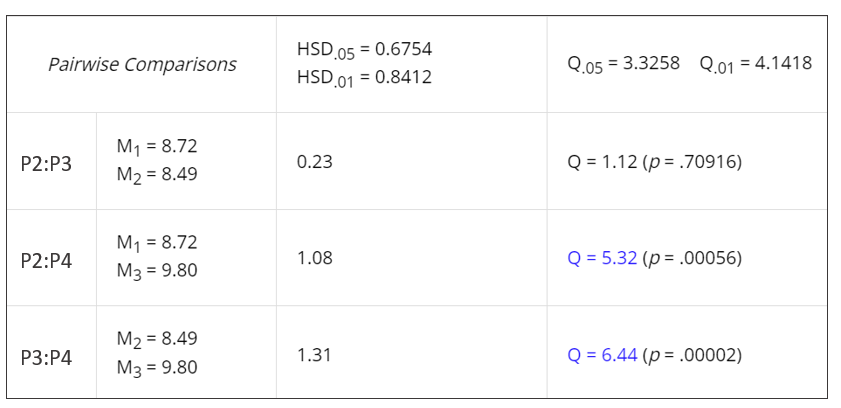
Supplement 4. HbA1c data by PEDIS analysed using an ANOVA with post hoc Tuckey to analyse pairwise comparisons within the ANOVA data. The F statistic informs if there is an overall difference between sample means. Tukey's test allows the determination between which of the various pairs of means - if any of them - there is a significant difference.

| **PEDIS Classification of both SST DFI + DFO with HbA1c measurement at baseline** | | | ***p-*Value** |
| --- | --- | --- | --- |
| **PEDIS 2**  **Mean HbA1C %** | **PEDIS 3**  **Mean HbA1C %** | **PEDIS 4**  **Mean HbA1C %** | ***p=*.0001**  **f value 9.04** |
| 8.7 | 8.5 | 9.8 |  |

Supplement 5. The contingency table below provides the following information: the observed cell totals, (the expected cell totals) and [the chi-square statistic for each cell].

|  | Resolved | Failed | Row Totals |
| --- | --- | --- | --- |
| SST-DFI | 268 (226.23) [7.71] | 58 (99.77) [17.48] | 326 |
| OM | 181 (222.77) [7.83] | 140 (98.23) [17.76] | 321 |
| Column Totals | 449 | 198 | 647 (Grand Total) |

The chi-square statistic is 50.7814. The p-value is < 0.00001. Significant at p < .05. The chi-square statistic with Yates correction is 49.5728. The p-value is < 0.00001. Significant at p < .05.

|  | Resolved | Failed | Row Totals |
| --- | --- | --- | --- |
| PEDIS 2 | 211 (168.26) [10.86] | 32 (74.74) [24.44] | 243 |
| PEDIS 3 | 211 (225.04) [0.88] | 114 (99.96) [1.97] | 325 |
| PEDIS 4 | 26 (54.70) [15.06] | 53 (24.30) [33.90] | 79 |
| Column Totals | 448 | 199 | 647 (Grand Total) |

The chi-square statistic is 87.1079. The p-value is < 0.00001. The result is significant at p < .05.

|  | Resolved | Failed | Row Totals |
| --- | --- | --- | --- |
| PEDIS 3 (O) | 171 (155.06) [1.64] | 104 (119.94) [2.12] | 275 |
| PEDIS 4 (O) | 10 (25.94) [9.79] | 36 (20.06) [12.66] | 46 |
| Column Totals | 181 | 140 | 321 (Grand Total) |

The chi-square statistic is 26.2101. The p-value is < .00001. The result is significant at p < .05.

Supplement 6. The contingency table below provides the following information: the observed cell totals, (the expected cell totals) and [the chi-square statistic for each cell].

|  | SST-DFI Resolved | SST-DFI Failure | Row Totals |
| --- | --- | --- | --- |
| PAD | 72 (84.67) [1.9] | 31 (18.33) [8.77] | 103 |
| No PAD | 196 (183.33) [0.88] | 27 (39.67) [4.05] | 223 |
| Column Totals | 268 | 58 | 326 (Grand Total) |

The chi-square statistic is 15.5895. The p-value is .000079. Significant at p < .05. The chi-square statistic with Yates correction is 14.3838. The p-value is .000149. Significant at p < .05.

|  | | | |
| --- | --- | --- | --- |
|  | OM Resolved | OM Failure | Row Totals |
| PAD | 71 (72.17) [0.02] | 57 (55.83) [0.02] | 128 |
| No PAD | 110 (108.83) [0.01] | 83 (84.17) [0.02] | 193 |
| Column Totals | 181 | 140 | 321 (Grand Total) |

The chi-square statistic is 0.0729. The p-value is .787187. Not significant at p < .05. The chi-square statistic with Yates correction is 0.024. The p-value is .876795. Not significant at p < .05.

Supplement 7. The contingency table below provides the following information: the observed cell totals, (the expected cell totals) and [the chi-square statistic for each cell].

| Ischemia Grade | SST-DFI Resolved | SST-DFI Failure | Row Totals |
| --- | --- | --- | --- |
| 0 | 196 (183.33) [0.88] | 27 (39.67) [4.05] | 223 |
| 1 | 43 (41.10) [0.09] | 7 (8.90) [0.40] | 50 |
| 2 | 14 (18.91) [1.27] | 9 (4.09) [5.89] | 23 |
| 3 | 15 (24.66) [3.79] | 15 (5.34) [17.49] | 30 |
|  |  |  |  |
| Column Totals | 268 | 58 | 326 (Grand Total) |

The chi-square statistic is 33.8558. The p-value is < 0.00001. The result is significant at p < .05.

| Ischemia Grade | OM Resolved | OM Failure | Row Totals |
| --- | --- | --- | --- |
| 0 | 110 (108.83) [0.01] | 83 (84.17) [0.02] | 193 |
| 1 | 39 (31.01) [2.06] | 16 (23.99) [2.66] | 55 |
| 2 | 15 (21.43) [1.93] | 23 (16.57) [2.49] | 38 |
| 3 | 17 (19.74) [0.38] | 18 (15.26) [0.49] | 35 |
|  |  |  |  |
| Column Totals | 181 | 140 | 321 (Grand Total) |

The chi-square statistic is 10.0351. The p-value is .01827. The result is significant at p < .05.
